# Benefits and Limitations to Plastic Mulching and Nitrogen Fertilization on Grain Yield and Sulfur Nutrition: Multi-Site Field Trials in the Semiarid Area of China

**DOI:** 10.3389/fpls.2022.799093

**Published:** 2022-02-22

**Authors:** Laichao Luo, Xiaoli Hui, Gang He, Sen Wang, Zhaohui Wang, Kadambot H. M. Siddique

**Affiliations:** ^1^State Key Laboratory of Crop Stress Biology in Arid Areas, Northwest A&F University, Yangling, China; ^2^Anhui Province Key Laboratory of Farmland Ecological Conservation and Pollution Prevention, School of Resources and Environment, Anhui Agricultural University, Hefei, China; ^3^The UWA Institute of Agriculture and School of Agriculture and Environment, The University of Western Australia, Perth, WA, Australia

**Keywords:** grain quality, sulfur accumulation, remobilization and post-uptake, soil availability sulfur, nitrogen regulation

## Abstract

Plastic mulching (PM) is widely used to improve crop water use efficiency and grain yield, but few studies have reported the effects of PM on cereal crop quality, especially sulfur (S) nutrition of wheat, which has significant effects on grain protein content, dough rheology, baking quality and human health. To fill this knowledge gap, we conducted a multi-site field experiment on the Loess Plateau from 2014 to 2016 to study the effects of PM combined with nitrogen (N) fertilizer on grain yield, shoot S accumulation, and grain S concentration of winter wheat in dryland. Compared with no mulching (NM), PM increased grain yield by 13.7% but decreased grain S concentration, S requirement for 1,000 kg^–1^ grain, soil available S concentration, and post-anthesis S uptake by 9.0, 9.7, 24.4, and 51.8%, respectively. Plastic mulching significantly increased shoot S accumulation at anthesis by 19.2%, but there was no significant difference at maturity. Additionally, grain S concentration and S requirement had a linear-plateau relationship with N fertilization amount, reaching maximum values at 110 and 127 kg N ha^–1^ under PM, 37.5 and 27.0% higher than those under NM. Furthermore, shoot S accumulation and N application rates well-fitted the linear-plateau model at anthesis and maturity. At maturity, straw, grain, and shoots accumulated the most S at threshold N rates of 120, 85 and 110 kg N ha^–1^, respectively. Crucially, stem + leaf S concentration at anthesis had a significant linear relationship with grain S concentration under PM; a 1 g kg^–1^ increase in stem leaf concentration corresponded with a 0.24 g kg^–1^ increase in grain S concentration. This study’s findings suggest that combining soil S supplementation with optimal N fertilizer under PM in northwest China and other regions with similar cropping systems increases grain S concentration and improves nutritional and processing qualities.

## Introduction

Sulfur (S) is an indispensable chemical element in the human body. Sulfur deficiency can cause frequent skin disease and alopecia and exacerbate the occurrence of blood glycosylation and cardiovascular diseases, threatening human health (Herr and Buchler, 2010; Ingenbleek and Kimura, 2013). The current per capita daily intake of S from wheat and starchy foods is 152.8 mg, accounting for 17.0% of total intake, higher than fruits, meat, and dairy products (Doleman et al., 2017). Therefore, it is vital to understand and evaluate the S nutrition status of cereal crops in countries or regions where wheat is the staple food.

Sulfur metabolism in cereal crops is closely related to nitrogen (N) metabolism, with efficient cysteine synthesis requiring both N and S (Zörb et al., 2013; Carciochi et al., 2020). Field experiments on wheat and maize crops have shown synergistic effects between N and S (Flæte et al., 2005; Hamnér et al., 2017; Xie et al., 2017; Tao et al., 2018), with a significant positive correlation between shoot N and S accumulation. Every 10 kg ha^–1^ increase in shoot N in wheat varieties with high and moderate N/S ratios increased S accumulation by 1.1 and 1.6 kg ha^–1^, respectively (Wang et al., 2009). Studies have also shown that the increasing involvement of amino acids in protein synthesis increases the activities of key enzymes for sulfate assimilation, increasing S flux through the sulfate assimilation pathway (Zhao et al., 1999; Scherer, 2001; Hawkesford and Kok, 2006). Under soil N deficiency, S remobilization in crop shoots can account for 39% of grain S accumulation (Wang et al., 2017). Increased N fertilizer significantly increased shoot S accumulation by 20.7–64.0% in wheat. Every 100 kg N ha^–1^ increase in N fertilizer decreased the proportion of grain S contributed from vegetative organs by 9.3% but increased the proportion of grain S contributed from post-anthesis S uptake by 9.3% (Wang and Yu, 2007). However, excessive N application does not increase root activity thus inhibiting root S uptake and reducing shoot S accumulation and grain S content (Wang and Yu, 2007; Xu et al., 2018).

Drylands account for about 80% of the world’s arable land, producing 60% of the world’s food (Singh et al., 2013). By the end of the 21st century, semiarid areas will have increased by 11–23%, with 78% of this increase in developing countries (Huang et al., 2016). Plastic mulching has been widely used for crop production in drylands to maximize the preservation of precipitation and improve crop water use efficiency (Khalil et al., 2014; Kader et al., 2017; Luo et al., 2018). Plastic mulching can increase the moisture storage capacity of dryland soil by 32–89 mm and crop water use efficiency by 0.2–19.5 kg ha^–1^ mm^–1^ (Wang et al., 2016; He et al., 2017; Zhu et al., 2018). At the same time, temperatures in the plow layer can increase by 0.98–10.0°C under PM, accelerating emergence and prolonging the growing period to promote grain development and grain filling (Li et al., 1999; Gan et al., 2013; Wang et al., 2014). In addition, PM significantly increases the number of soil microbes, with bacteria and ammonifier counts increasing by 16.3 and 12.9%, respectively, promoting the activation of soil nutrients and their effective uptake by crops and thus improving nutrient use efficiency and crop yield (Steinmetz et al., 2016; Kader et al., 2017; Zhu et al., 2018).

Drylands in northwest China are typical rainfed farming regions, with a total planting area of 160 million ha (Li and Xiao, 1992). This region is subject to low and unevenly distributed precipitation, mainly concentrated in the summer fallow season. Plastic mulching has a significant promoting effect, increasing wheat yields by 13.7–50.7% (Li et al., 1999; Luo et al., 2018). During wheat growth, the soil moisture status affects shoot S uptake and utilization (Gooding et al., 2003; Zhang et al., 2011). Increased soil moisture storage capacity during jointing or anthesis in winter wheat increased ear S concentration by 51.3% and grain S concentration by 16.4% (Zhang et al., 2011; Liu et al., 2014). Similarly, maize experiments on the North China Plain also showed that during the grain-filling period, sufficient water and sulfur supply resulted in a significant increase in both water productivity at the grain yield level and grain yield (Ma et al., 2021). It is well-known that PM changes the soil moisture status and improves crop yield in dryland areas. However, there are no systematic reports on the effect of PM on S uptake, utilization, or concentration in crops, or the effect of N rate on wheat S nutrition. Therefore, multi-site field experiments were conducted in a typical dryland area of the Loess Plateau to investigate the efficacy of PM and N rate on grain yield and S nutrition of winter wheat. The study aimed to (1) evaluate the effect of PM on grain yield and S nutrition of winter wheat, particularly grain S concentration, S requirement for 1,000 kg^–1^ grain, and shoot S accumulation and remobilization, (2) clarify and quantify the relationship between shoot S nutrition and N fertilization under PM, and (3) forecast grain S concentration based on shoot S concentration at anthesis. This information will provide a scientific basis for improving the nutrition status related to dietary S of cereal crops in semiarid areas.

## Materials and Methods

### Experimental Sites

**FIGURE 1:**
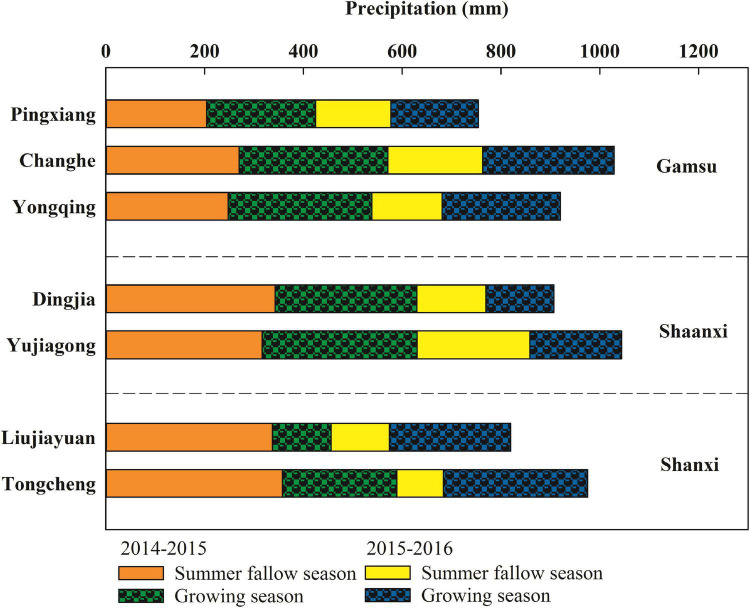
Precipitation distribution at each experimental site for 2014–2015 and 2015–2016. Precipitation data for Tongcheng, Liujiayuan, Yujiagong, and Dingjia extracted from the rain gauge (7852 Rain Collector II, Onset Computer Corporation, United States) installed near each site. Precipitation data for Yongqing, Changhe, and Pingxiang in Gansu Province obtained from the China Meteorological Data Service Center (CMDC).

The field experiment was carried out at seven sites in three provinces of the main winter wheat planting region on the Loess Plateau in the 2014–2015 and 2015–2016 cropping seasons. The sites were Tongcheng and Liujiayuan in Shanxi Province, Yujiagong and Dingjia in Shaanxi Province, and Yongqing, Changhe, and Pingxiang in Gansu Province (34°43′–36°23′ N, 107°07′–111°35′ E), with altitudes ranging from 500 to 1760 m. A monocropping system (winter wheat–summer fallow) is used in this region. The average annual precipitation is 550 mm, and the precipitation from July to September accounts for 60–70% of the annual total. The distribution of precipitation during the winter wheat growing season and the summer fallow season at each experimental site is in Figure 1. The soil at each experimental site was loam; the physiochemical properties of the soil before sowing are in Table 1.

**TABLE 1:** Initial physical and chemical characteristics of the topsoil (0–20 cm) at each experimental site.

Parameters	Shanxi Province		Shaanxi Province		Gansu Province		
	Tongcheng	Liujiayuan	Yujiagong	Dingjia	Yongqing	Changhe	Pingxiang
Organic C (g kg^–1^)	7.8	10.8	10.2	8.5	7.9	6.1	7.7
Total N (g kg^–1^)	0.84	0.80	0.75	0.77	0.90	0.95	0.93
NO_3_^–^-N (mg kg^–1^)	11.5	22.6	3.5	13.1	16.8	12.2	5.5
NH_4_^+^-N (mg kg^–1^)	1.1	0.4	0.3	2.6	0.8	0.5	0.3
Olsen-P (mg kg^–1^)	17.6	10.7	13.4	4.5	12.0	11.3	12.5
Available K (mg kg^–1^)	160.0	202.4	83.9	130.0	102.2	122.7	117.3
Available S (mg kg^–1^)	46.7	36.7	20.0	9.9	2.1	2.5	3.0
pH (H_2_O)	8.1	7.9	8.5	8.2	8.4	8.6	8.7

### Experiment Design and Field Agronomic Management

The field experiment was based on a split-plot design, with cultivation system as the main plot (no mulching and plastic mulching). No mulching is the local cultivation pattern, which involves conventional flat planting and machine drilling with 20 cm row spacing. After wheat harvest, the straw is removed, leaving the farmland surface bare during the summer fallow season. Details on the specific operation of PM are described in Luo et al. (2019). The subplots were treated with N fertilizer at 0, 60, 120, 180, and 240 kg N ha^–1^. Nitrogen, phosphorus (60–150 kg P_2_O_5_ ha^–1^), and potassium (only in Liujiayuan, 37.5 kg K_2_O ha^–1^) fertilizers with urea (N 46%), calcium superphosphate (P_2_O_5_ 12%) and potassium chloride (K_2_O 60%) were applied as basal fertilizer, with a one-time application with rotary tillage before sowing. The seeding rates (90–270 kg ha^–1^) and seeding dates (1 Oct.–20 Sep.) are adjusted by the temperature, precipitation and soil moisture content in different experimental sites to get the best state. There was no irrigation applied, and other field management processes were consistent with local farmer practices.

### Soil and Plant Sampling and Analysis

At anthesis, surface soil to 20 cm depth was randomly sampled from five points in each plot, with the samples extracted using the quartering method (Bao, 2000). Soil available S concentration was determined using barium sulfate turbidimetry and ultraviolet spectrophotometer (UV-2450, Shimadu, Japan).

Plant samples were collected at anthesis and maturity (GS 61 and 92); the sampling dates for each site are in Supplementary Table 1. On both sampling dates, plants of 100 ears were randomly selected from each plot, and the roots cut from the shoots. At maturity, shoot samples were separated into straw and grain. A 50 g random subsample from the main sample was washed with tap water two to three times, cleaned with ultrapure water three times, and dried at 75°C to constant weight, and weighed. The dried straw at anthesis and straw and grain samples at maturity were smashed by a ball mill (MM410, Retsch, Germany) and digested with a high-throughput microwave-digestion apparatus (Multiwave PRO, Anton Paar, Austria) using the HNO_3_-H_2_O_2_ method (Matusiewicz et al., 1989). The S concentration in the digestion solution was determined by ICP-MS (ICAP Qc, Thermo Fisher Scientific, United States). For each batch of digestion samples, standard samples (GBW10011–wheat) were included, which were treated and measured using the same procedure to validate the protocol.

For grain yield calculation, plants were manually collected from four 1 × 1 m quadrats in each plot, air-dried, mechanically threshed, and weighed. About 150 g of grain per sample was dried and weighed to calculate grain moisture content. Shoot biomass and grain yield were expressed as dry weight.

### Statistical Analysis

Based on Graß et al. (2013) and Kozak and Piepho (2018), the data was examined for normality and homoscedasticity; a mixed model option in SAS 9.2 was used for further analysis. When the ANOVA results (*F*-values) were significant, least significant differences (LSD) between mean values were determined at *P* < 0.05. Regression analysis was conducted by using the average value of each N application rate plot under NM and PM in Yujiagong of Shaanxi Province during two cropping seasons, and according to the criteria (lowest residual sum of square and largest R2) to determine the best-fit equations, such as linear, quadratic or linear-plateau models. Linear and non-linear procedure were carried out using SAS and SigmaPlot systems to plot curves that best-fit the parameters response to N rate, and the relationships between grain S concentration at maturity and stem + leaf or ear S concentration at anthesis of winter wheat in dryland.

## Results

### Grain Yield, Sulfur Concentration, and Sulfur Requirement for 1,000 kg^–1^ Grain

**FIGURE 2:**
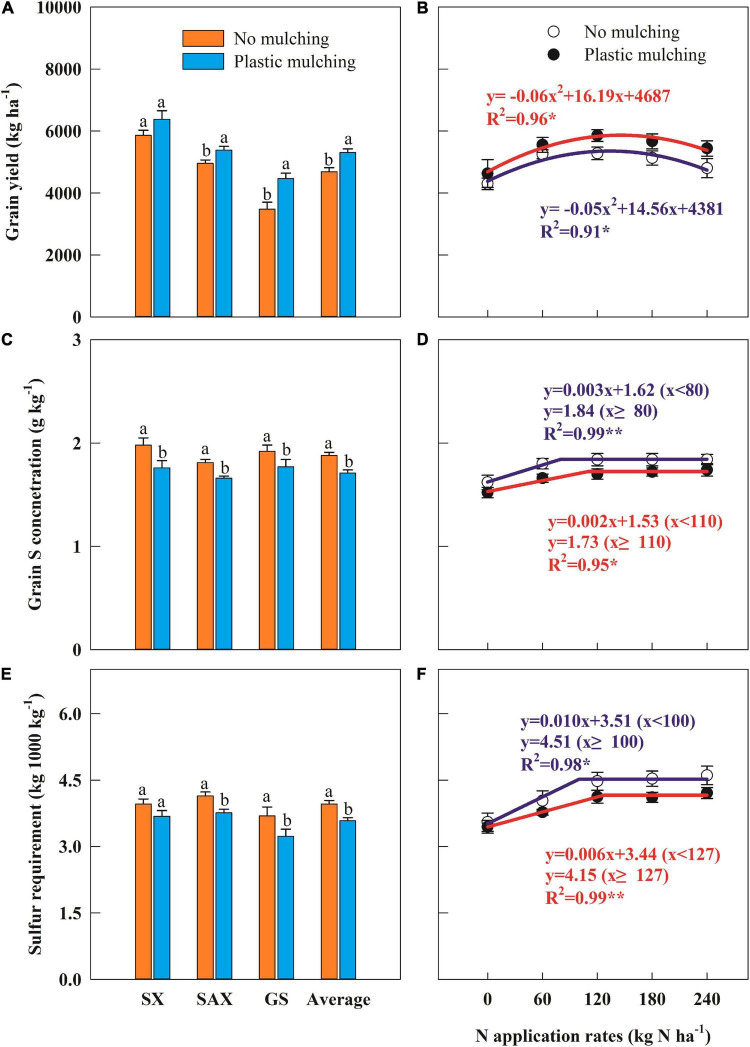
Impact of plastic mulching and N application rate on grain yield **(A,B)**, grain sulfur (S) concentration **(C,D)**, and grain S requirement **(E,F)** of winter wheat in dryland during two cropping seasons (2014–2015 and 2015–2016). SX, Shanxi; SAX, Shaanxi; GS, Gansu. Error bars represent standard errors of replications. Different lowercase letters indicate significant differences (*P* < 0.05) between plastic mulching and no mulching. *P < 0.05; **P < 0.01.

Across all sites and years, winter wheat grain yields averaged 5,246 kg ha^–1^ under PM, 13.7% higher than under NM (Figure 2A). Wheat yields at the experimental sites in the Shaanxi and Gansu Provinces significantly increased under PM by 8.5 and 28.6%, respectively, but there was no significant difference in Shanxi Province. The increase in wheat yields under PM varied significantly between years (Supplementary Table 2). Grain yield varied with N rate in a negative quadratic model, with maximum yields of 5,261 kg ha^–1^ at 121 kg N ha^–1^ under NM and 5,863 kg ha^–1^ at 146 kg N ha^–1^ under PM (Figure 2B).

Grain S concentration and S requirement declined significantly under PM compared to NM, by 9.0 and 9.7% averaged across sites and years (Figures 2C,E and Supplementary Table 2). Grain S concentration and S requirement also varied with N rate in the linear-plateau model (Figures 2D,F). The regression analysis revealed that grain S concentration plateaued at 1.84 g kg^–1^ with 80 kg N ha^–1^ (critical N rate) under NM and 1.73 g kg^–1^ with 110 kg N ha^–1^ under PM. Similarly, the S requirement plateaued at 4.51 kg 1,000 kg^–1^ grain with 100 kg N ha^–1^ under NM and 4.15 kg 1,000 kg^–1^ grain with 127 kg N ha^–1^ under PM.

### Soil Available Sulfur Concentration

**FIGURE 3:**
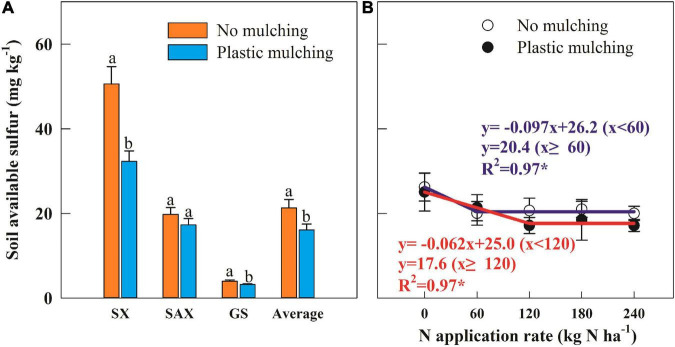
Impact of plastic mulching **(A)** and N application rate **(B)** on soil available sulfur in the topsoil layer (0–20 cm) during two cropping seasons (2014–2015 and 2015–2016). SX, Shanxi; SAX, Shaanxi; GS, Gansu. Error bars represent standard errors of replications. Different lowercase letters indicate significant differences (*P* < 0.05) between plastic mulching and no mulching. *P < 0.05.

The soil available S concentration in the 0–20 cm soil layer decreased under PM compared to NM, by 24.4% averaged across sites and years, with average declines of 36.2% in Shanxi Province and 20.0% in Gansu Province (Figure 3A) and 9.6% in both years (Supplementary Figures 1A,B), but no significant variation in Shaanxi Province. Soil available S concentration also varied with N rate in a negative linear-plateau model (Figure 3B). The regression analysis revealed critical N rates of 60 and 120 kg N ha^–1^, with minimum soil available S concentrations of 20.4 mg kg^–1^ under NM and 17.6 mg kg^–1^ under PM.

### Shoot Sulfur Accumulation

**FIGURE 4:**
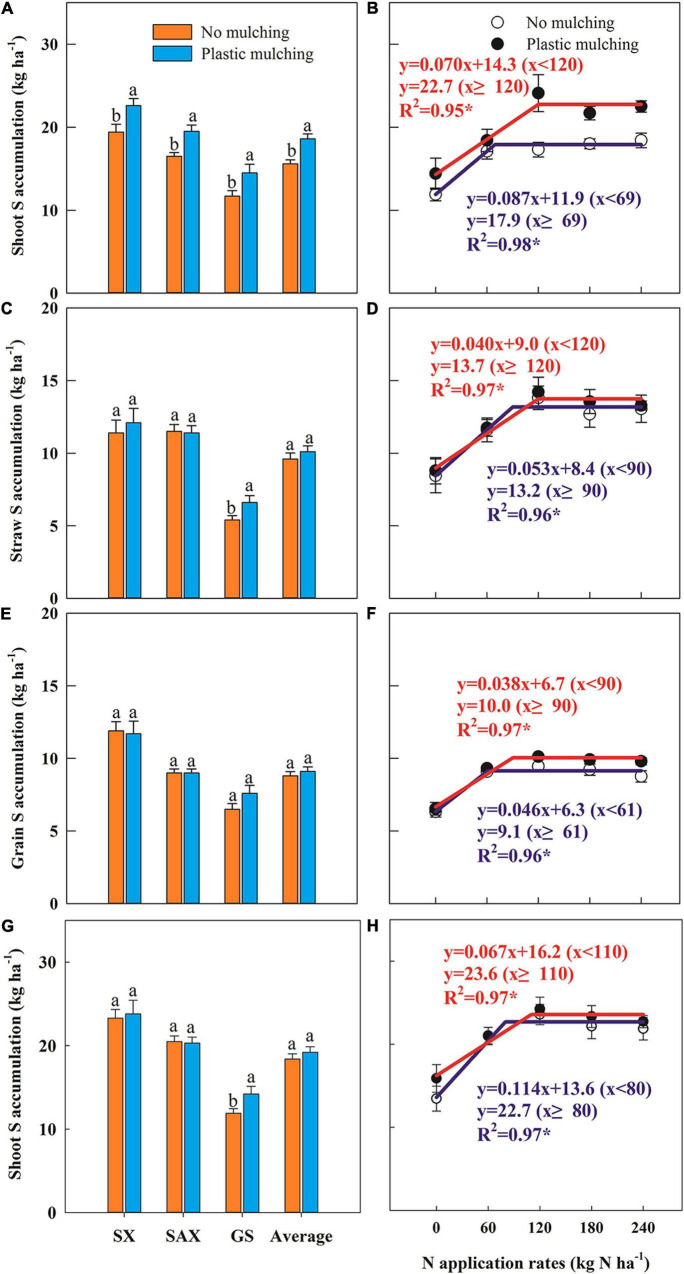
Impact of plastic mulching and N application rate on shoot sulfur (S) accumulation at anthesis **(A,B)**, straw S accumulation **(C,D)**, grain S accumulation **(E,F)**, and shoot S accumulation at maturity **(G,H)** of winter wheat in dryland during two cropping seasons (2014–2015 and 2015–2016). SX, Shanxi; SAX, Shaanxi; GS, Gansu. Error bars represent standard errors of replications. Different lowercase letters indicate significant differences (*P* < 0.05) between plastic mulching and no mulching. *P < 0.05.

Shoot S accumulation at anthesis increased under PM compared to NM, by 19.2% averaged across sites and years, with average increases of 16.2, 18.4, and 24.1% in Shanxi, Shaanxi, and Gansu Provinces, respectively (Figure 4A). Shoot S accumulation varied significantly between years, increasing by 22.5% in 2015 and 16.2% in 2016 under PM compared to NM (Supplementary Figures 1C,D). Shoot S accumulation at anthesis also varied with N rate in a linear-plateau model (Figure 4B), with a critical N rate of 120 kg N ha^–1^ under PM, 73.9% higher than under NM.

Straw, grain, and shoot S accumulation at maturity did not significantly increase under PM, averaged across sites and years (Figures 4C,E,G and Supplementary Figure 2). The S accumulation responses in straw, grain, and shoots under PM fitted a positive linear-plateau model, with critical N rates of 120, 90, and 110 kg N ha^–1^, respectively, being 33.3, 47.5, and 37.5% higher than under NM (Figures 4D,F,H).

### Sulfur Remobilization and Uptake During Grain Filling

**FIGURE 5:**
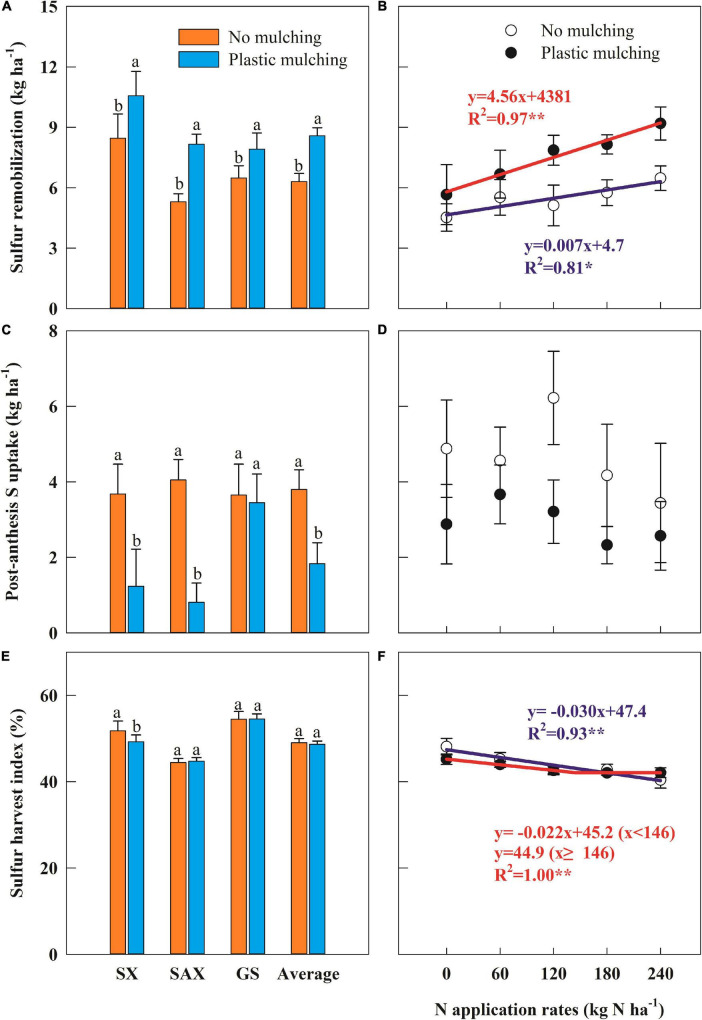
Impact of plastic mulching and N application rate on sulfur (S) remobilization **(A,B)**, post-anthesis S uptake **(C,D)**, and S harvest index **(E,F)** of winter wheat in dryland during two cropping seasons (2014–2015 and 2015–2016). SX, Shanxi; SAX, Shaanxi; GS, Gansu. Error bars represent standard errors of replications. Different lowercase letters indicate significant differences (*P* < 0.05) between plastic mulching and no mulching. *P < 0.05; **P < 0.01.

Compared to NM, S remobilization averaged across sites and years increased by 36.0% under PM (Figure 5A). The results varied between sites and years (Supplementary Figures 3A,B), with PM increasing S remobilization by 24.9% in Shanxi, 54.0% in Shaanxi, and 22.0% in Gansu Provinces, and by 49.6% in 2015 and 24.9% in 2016. Moreover, S remobilization increased linearly with N rate; every 1 kg N ha^–1^ increase had a corresponding 4.6 kg ha^–1^ increase in S remobilization (Figure 5B).

Conversely, post-anthesis S uptake averaged across sites and years declined by 51.8% under PM compared to NM (Figure 5C). Post-anthesis S uptake under PM declined on average by 66.6% in Shanxi and 80.0% in Shaanxi, with no significant change in Gansu. Post-anthesis S uptake did not correlate with increasing N rate (Figure 5D). Additionally, the PM effect on S harvest index was not significant, although it declined by 4.9% in Shanxi, relative to NM (Figure 5E). The S harvest index under PM varied with N rate in a linear-plateau model (Figure 5F), with a critical N rate of 146 kg N ha^–1^.

### Relationship Between Organs Sulfur Concentrations at Anthesis and Grain Sulfur Concentration

**FIGURE 6:**
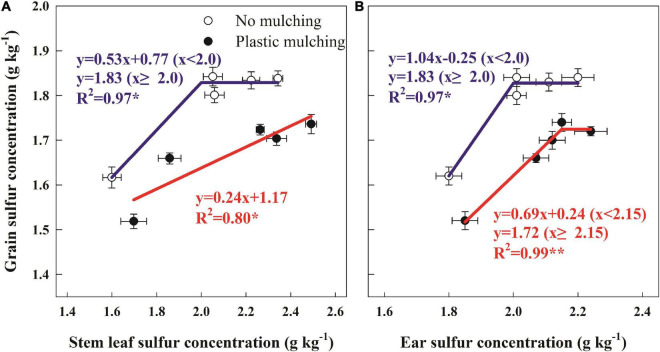
Regressions between grain sulfur (S) concentration and stem + leaf S concentration at anthesis **(A)**, grain S concentration, and ear S concentration at anthesis **(B)** of winter wheat in dryland under plastic mulching during two cropping seasons (2014–2015 and 2015–2016). *P < 0.05; **P < 0.01.

The increase in stem + leaf S concentration under PM at anthesis significantly increased grain S concentration; every 1 g kg^–1^ increase in stem + leaf S concentration corresponded to a 0.24 g kg^–1^ increase in grain S concentration (Figure 6A). Moreover, grain S concentration had a linear-plateau relationship with ear S concentration (Figure 6B). Under PM, the threshold ear S concentration before grain S concentration reached its maximum (plateau at 1.72 g kg^–1^) was 2.15 g kg^–1^.

## Discussion

### Grain Yield Affected by Plastic Mulching

The grain yield of winter wheat under PM in the three Provinces increased on average by 13.7% but was more pronounced in Gansu Province (58.6%) (Figure 2A). Plastic mulching increases the temperature of the plow layer, reduces soil moisture loss during crop growth, increases radiation and water use efficiencies, produces more photoassimilates, and increases source and sink size during crop growth, increasing crop yield (He et al., 2017; Ding et al., 2021). The increase in crop yield is better understood from the perspective of crop yield components. Field studies in northwest China, Kenya, Pakistan, and Australia showed that PM increased spike number by 27.2–56.6%, and thus wheat yield by 73.4–163.3% (Hu et al., 2012; Wang et al., 2016; Daryanto et al., 2017; Javed et al., 2018). The present study revealed that the increased wheat yield under PM was significantly related to a 9.2% increase in spike number (Supplementary Figure 4). Similar results have been reported for grain yield and its components of wheat in other regions of China and East Africa (Wang et al., 2016; Zhang et al., 2018).

### Grain Sulfur Concentration Affected by Plastic Mulching

Compared to NM, average shoot S accumulation at anthesis increased by 19.2%, indicating that PM had a positive promoting effect on shoot S accumulation at anthesis (Figure 4A). Most studies report that PM improved soil moisture and nutrient conditions, activating soil nutrients (Kader et al., 2017; Zhu et al., 2018). Meanwhile, PM promoted root development, increasing root surface area density, root diameter, and root dry weight by 45.4, 32.5 and 27.7%, respectively, due to increased soil water storage, root volume density (33.3–50.0%), and root weight density (42.7–68.8%) at 50–100 days after planting, promoting nutrient uptake and utilization and crop growth and development (Jia et al., 2018). Isotope labeling experiments with ^34^S revealed that 14% of grain S originated from shoot S accumulation at the emergence–jointing stage, 30% at the jointing–flagging stage, and 6% at the flagging–anthesis stage, suggesting that pre-anthesis S accumulation accounted for 50% of grain S (Monaghan et al., 1999). Similarly, Wang and Yu (2007) reported that leaf and ear S accumulation at anthesis had significant positive correlations with the amount of S remobilization, with every 1 mg increase in leaf or ear S accumulation corresponding to a 0.17 or 0.12 mg increase in the amount of S remobilization, respectively. It has also been suggested that higher soil available S concentration from anthesis to maturity period can increase the activity and content of ascorbate-glutathione antioxidant cycle-related enzymes in flag leaves, thus delaying the senescence of functional leaves of crops by improving antioxidant capacity (Lewandowska and Sirko, 2008; Zhu et al., 2010). Therefore, when using PM to improve soil moisture and temperature, pre-anthesis S accumulation does not limit grain S accumulation; instead, it likely strengthens grain S accumulation by serving as a source of S during post-anthesis S remobilization to grain. Shoot S remobilization increased by 36.0% under PM compared to NM, positively affecting grain S accumulation (Figure 5A).

In this study, PM did not significantly affect grain S accumulation in winter wheat at maturity (Figure 4E). Other studies have argued that reductions in grain S concentration resulted from a dilution effect due to the increase in wheat yield. However, our analysis indicated that the dilution effect accounted for only 46.3% of the change in grain S concentration; this raises the question, why did grain S uptake does not increase with wheat yield (Supplementary Figure 5). Post-anthesis S uptake resulted in higher amounts of S remobilization under PM, but significantly lower than post-anthesis S uptake under NM, being only 0.62 kg ha^–1^ or 77.9% lower than NM (Figure 5C). Under NM, post-anthesis S uptake contributes more than 50% to grain S (Hocking, 1994; Monaghan et al., 1999). However, only 5.4% of grain S originated from post-anthesis S uptake under PM, suggesting that PM reduced S uptake during grain filling and was the leading cause of the decline in grain S concentration. As Maillard et al. (2015) shows, this may also be related to the rapid remobilization of S from mature organs to young tissues or grains under the condition of S deficiency, and the accelerated aging process of organs, so that there is no significant S accumulation in grains at maturity stage.

In addition, available soil S is crucial for providing S nutrition to crops and promoting their growth and development. In northeast China, long-term PM for 18 consecutive years reduced the available S concentration in the plow soil layer by 4.3–32.7%, compared to NM (Wang et al., 2008). However, significant positive correlations occurred between shoot S concentration in wheat and available soil S concentration in China, Turkey, Australia, and other regions (Anderson et al., 2013; Zhao et al., 2014; Gülüt and Hoşgökdelen, 2021). Therefore, the lower available soil S during the grain filling stage may explain the decrease in post-anthesis S uptake of wheat under PM. Consequently, it is important to increase soil S supplementation under PM to improve S nutrition and the nutritional and processing qualities of wheat grain in dryland areas.

### Grain Sulfur Concentration Affected by Nitrogen Rate

The uptake and assimilation of S by crops is related to soil N level (Gallejones et al., 2012; Li et al., 2019). Sulfur in wheat grain is mostly in the form of cysteine and methionine, precursors of other S-containing compounds (Zhao et al., 1999; Nakamura et al., 2009). The carbon skeleton and N in cysteine are derived from serine, while N assimilation to serine occurs via the glutamine synthetase-glutamate synthase (GS-GOGAT) pathway (Shewry and Tatham, 1997; Zhao et al., 1999). Nitrogen levels regulate the affinity of S transporters and the number of assimilation precursors, which in turn affects the synthesis of S-containing amino acids and proteins in crops and the composition of grain proteins, ultimately affecting grain processing properties (Scherer, 2001). Salvagiotti and Miralles (2008) found that every 1 kg N ha^–1^ increase in N fertilizer resulted in a 23.5 and 38.4 kg ha^–1^ increase in shoot biomass of bread wheat under the S fertilizer of 5 and 35 kg S ha^–1^, respectively. Sulfur uptake by crops on calcareous soils also depends on the amount of N fertilizer applied, with high-N conditions causing crop S deficiency and other adverse effects related to the formation and development of crop reproductive organs (Zhao et al., 2014). A culture experiment on spring wheat in Norway revealed a quadratic relationship between shoot S concentration and N rate (200–300 kg N ha^–1^); high application rates induced overexpression of the serine proteinase inhibitor and deterioration of storage protein quality, affecting processing quality (Flæte et al., 2005). Wang and Yu (2007) confirmed that shoot S concentration and accumulation in wheat at maturity increased with increasing N rate to a peak before declining (0–285 kg N ha^–1^). The present study showed that shoot S accumulation had a linear-plateau relationship with the amount of N fertilizer applied (0–240 kg N ha^–1^) (Figure 4). Excessive N fertilizer application failed to promote S uptake, accumulation, and translocation in vegetative shoot organs, which was not conductive to S redistribution, reducing the contribution of S remobilization to grain and decreasing post-anthesis S uptake by winter wheat under PM. Therefore, it is necessary to strengthen the supplement of S fertilizer while optimally applying nitrogen to achieve the comprehensive nutrient management based on the pattern of high yield and high quality of cereals and continuous mono-cropping in drylands.

## Conclusion

Plastic mulching and N rates significantly affected grain yield, grain S concentration, and S requirement for 1,000 kg^–1^ grain in winter wheat grown in drylands. Compared with NM, PM significantly increased grain yield, but reduced grain S concentration and the S requirement for 1,000 kg^–1^ grain due to a decline in soil available S and post-anthesis S uptake. Grain S concentration, S requirement for 1,000 kg^–1^ grain, soil available S concentration, and shoot S accumulation had a linear-plateau relationship with N application rate, with more N input required under PM than NM. In addition, grain S concentration under PM could be predicted using the S concentration of vegetative organs at anthesis; every 1 g kg^–1^ increase in stem + leaf S concentration at anthesis corresponded to a 0.24 g kg^–1^ increase in grain S concentration.

## Data Availability Statement

The original contributions presented in the study are included in the article/Supplementary Material, further inquiries can be directed to the corresponding author.

## Author Contributions

ZW conceived and designed the study. GH and LL performed the experiments. LL and XH analyzed, interpreted the data, and involved in statistical analysis and drafting of the manuscript. ZW and KS critically revised the manuscript for important intellectual content. All authors have read and approved the final manuscript.

## Conflict of Interest

The authors declare that the research was conducted in the absence of any commercial or financial relationships that could be construed as a potential conflict of interest.

## Publisher’s Note

All claims expressed in this article are solely those of the authors and do not necessarily represent those of their affiliated organizations, or those of the publisher, the editors and the reviewers. Any product that may be evaluated in this article, or claim that may be made by its manufacturer, is not guaranteed or endorsed by the publisher.
