# Supplementary material for: Benefits and Limitations to Plastic Mulching and Nitrogen Fertilization on Grain Yield and Sulfur Nutrition: Multi-Site Field Trials in the Semiarid Area of China
Source: Front Plant Sci. 2022 Feb 22;13:799093. doi: 10.3389/fpls.2022.799093 (PMC8902349; doi:10.3389/fpls.2022.799093)
Supplement: Supplementary file 1 [file Presentation_1.pdf]

TABLE S1

Anthesis and harvest date at each experimental site in the two cropping years (2014–2015 and 2015–2016).

Province	Site	Sampling date-Anthesis		Sampling date-Harvest	
		2015	2016	2015	2016
Shanxi	Tongcheng	2 May	1 May	2 Jun.	3 Jun.
	Liujiayuan	4 May	5 May	7 Jun.	12 Jun.
Shaanxi	Yujiagong	11 May	11 May	17 Jun.	13 Jun.
	Dingjia	15 May	15 May	1 Jul.	21 Jun.
Gansu	Yongqing	28 May	27 May	25 Jun.	27 Jun.
	Changhe	30 May	6 Jun.	25 Jun.	10 Jul.
	Pingxiang	4 Jun.	10 Jun.	10 Jul.	15 Jul.

TABLE S2

Impact of plastic mulching on grain yield, grain S concentration, and grain S requirement of winter wheat in dryland during the two cropping years of 2014–2015 and 2015–2016.

Year	Province	Grain yield (kg ha^{-1})		Grain S concentration (g kg^{-1})		Grain S requirement (kg 1000 kg^{-1} grain)	
		No mulching	Plastic mulching	No mulching	Plastic mulching	No mulching	Plastic mulching
2014–2015	Shanxi	6154±265b	7212±345a	1.96±0.10a	1.79±0.11b	4.32±0.15a	3.93±0.21a
	Shaanxi	4582±124b	5292±145a	1.78±0.02a	1.63±0.04b	4.18±0.13a	3.75±0.14b
	Gansu	4552±205a	4687±298a	1.68±0.07a	1.49±0.10b	2.76±0.14a	2.52±0.14a
	Average	4894±136b	5498±186a	1.79±0.03a	1.62±0.04b	3.77±0.13a	3.41±0.12b
2015–2016	Shanxi	5559±136a	5541±234a	2.07±0.09a	1.80±0.09b	3.60±0.07a	3.43±0.11b
	Shaanxi	5335±145a	5486±203a	1.83±0.05a	1.68±0.03b	4.09±0.14a	3.77±0.09b
	Gansu	2400±102b	4251±184a	2.16±0.04a	1.96±0.03b	4.61±0.14a	3.93±0.12a
	Average	4482±217b	5110±147a	1.98±0.04a	1.79±0.03b	4.15±0.10a	3.75±0.07b

Different lowercase letters indicate significant differences (P<0.05) between plastic mulching and no mulching.

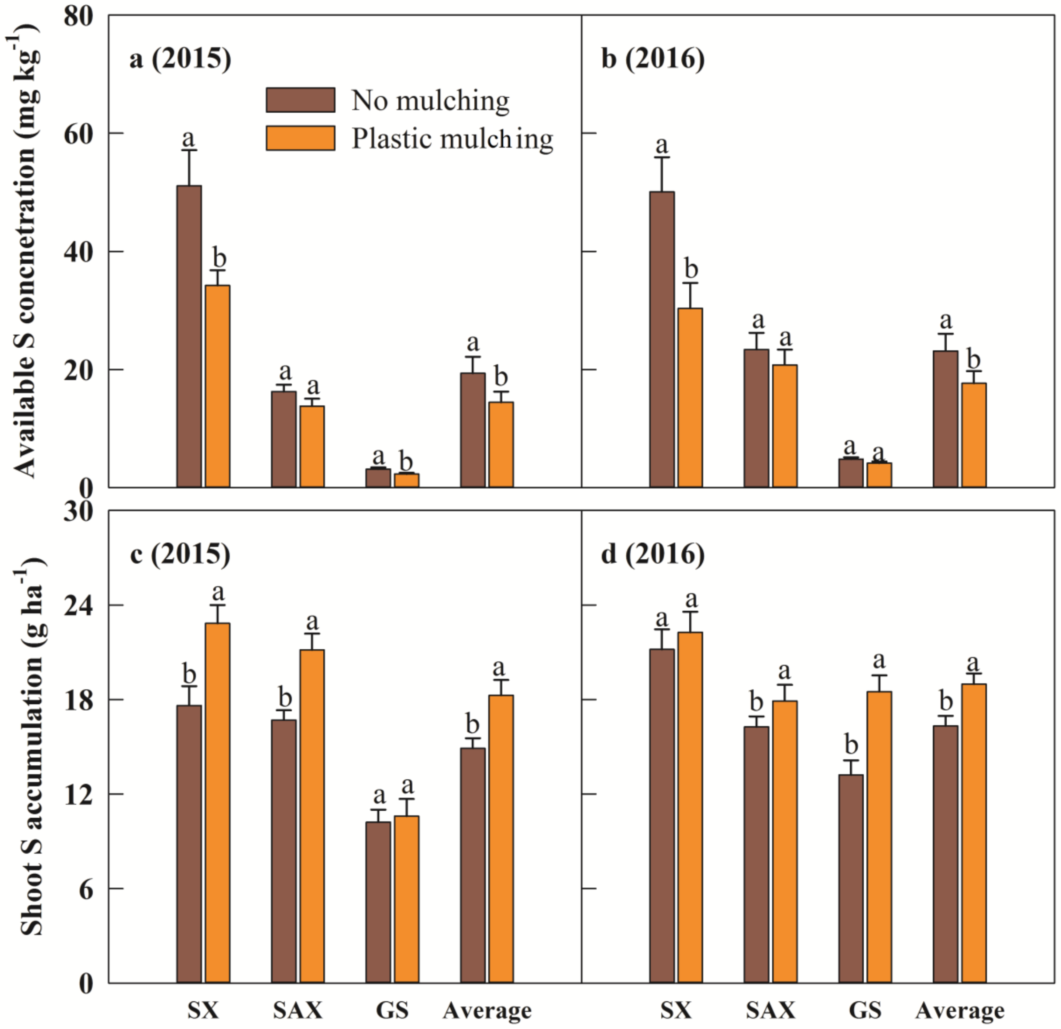

FIGURE S1 Impact of plastic mulching on soil available concentration (a, b) and shoot S accumulation (c, d) of winter wheat at anthesis during the two cropping years of 2014–2015 and 2015–2016. SX: Shanxi, SAX: Shaanxi, GS: Gansu. Different lowercase letters indicate significant differences (P<0.05) between plastic mulching and no mulching.

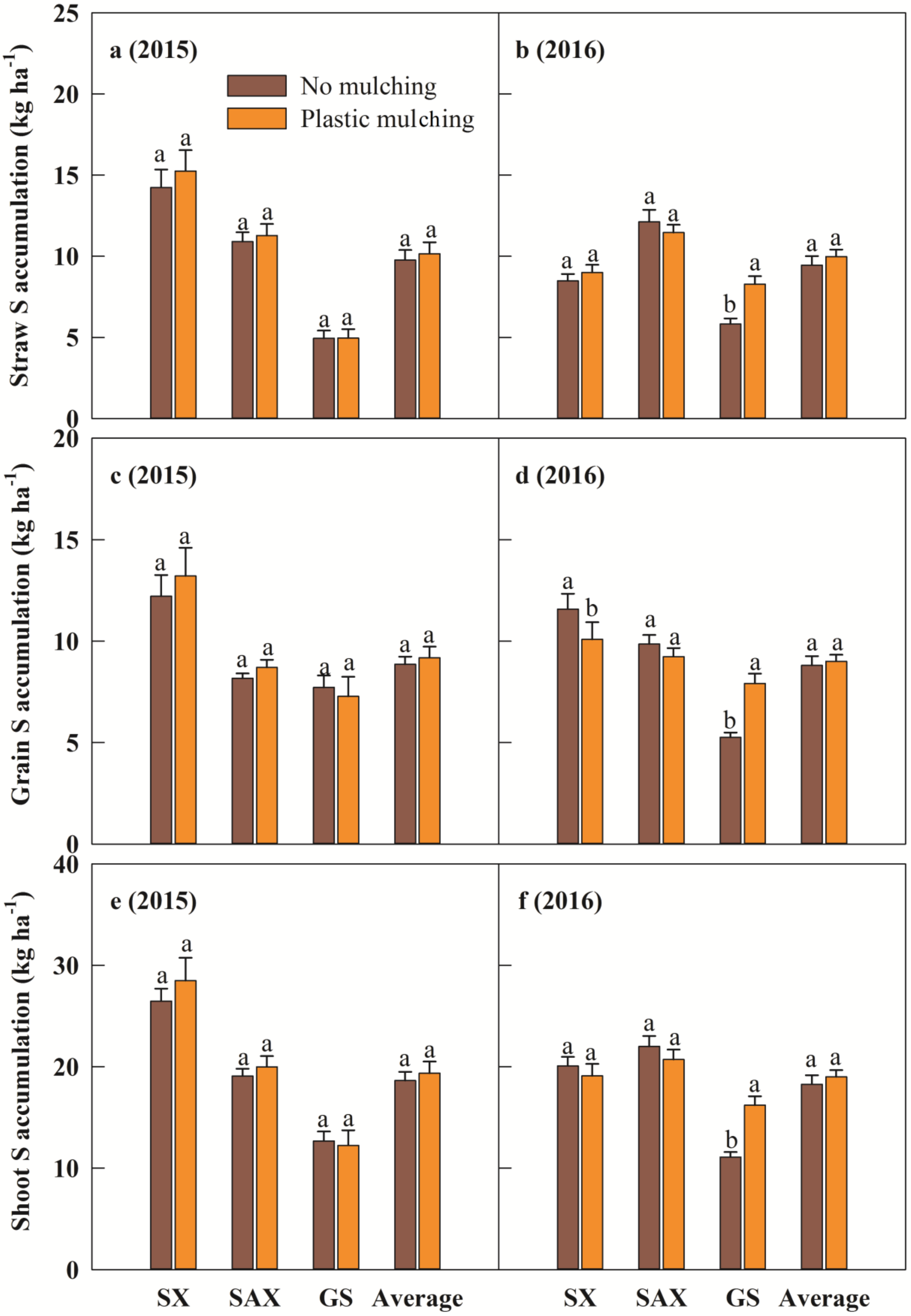

FIGURE S2 Impact of plastic mulching on straw S accumulation (a, b), grain S accumulation (c, d), and shoot S accumulation of winter wheat at maturity during the two cropping years of 2014–2015 and 2015–2016. SX: Shanxi, SAX: Shaanxi, GS: Gansu. Different lowercase letters indicate significant differences (P<0.05) between plastic mulching and no mulching.

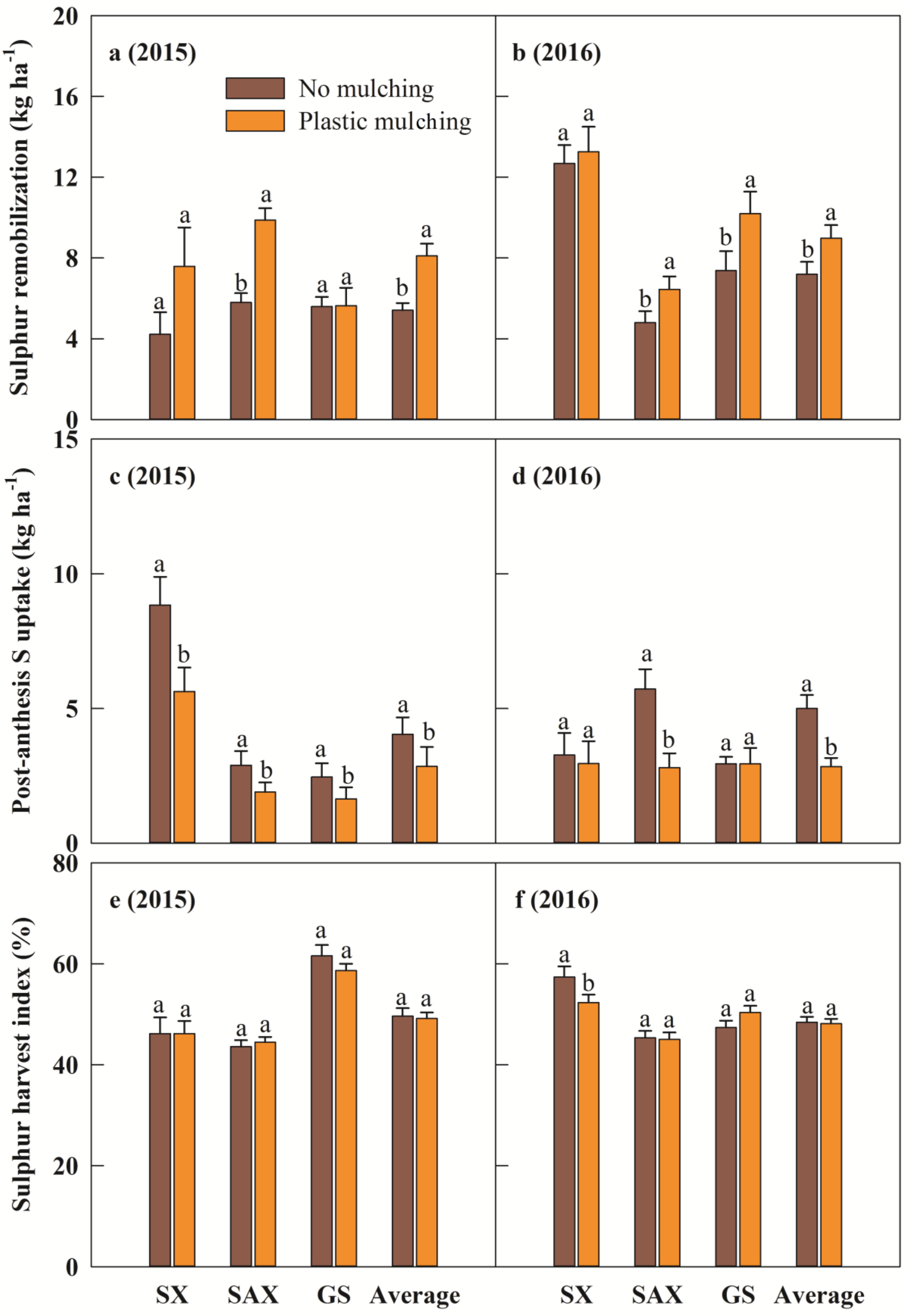

FIGURE S3 Impact of plastic mulching on S remobilization (a, b), post-anthesis S uptake (c, d), and S harvest index of winter wheat at maturity during the two cropping years of 2014–2015 and 2015–2016. SX: Shanxi, SAX: Shaanxi, GS: Gansu. Different lowercase letters indicate significant differences (P<0.05) between plastic mulching and no mulching.

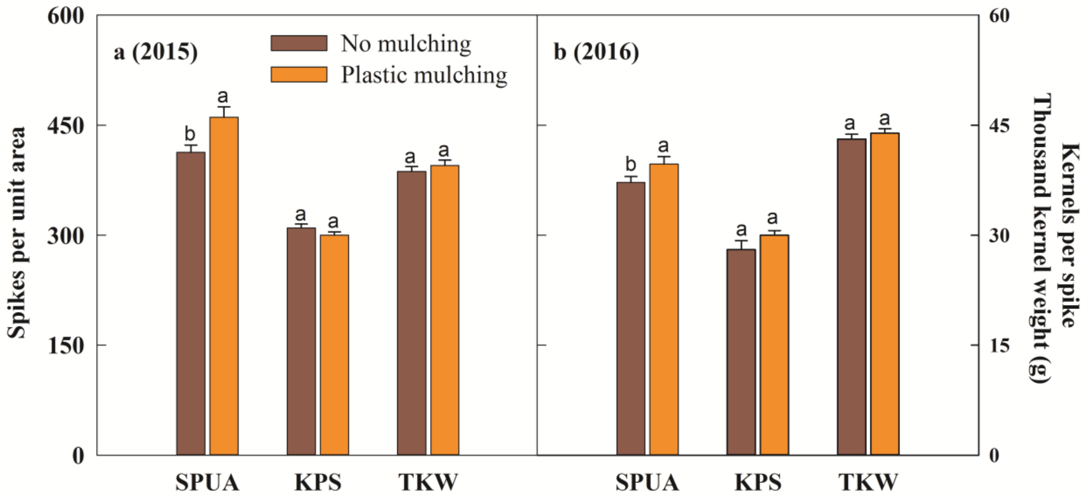

FIGURE S4 Impact of plastic mulching on spikes per unit area (SPUA), kernel per spike (KPS), and thousand kernel weight (TKW) of winter wheat in dryland during the two cropping years of 2014–2015 and 2015–2016. Different lowercase letters indicate significant differences (*P*<0.05) between plastic mulching and no mulching.

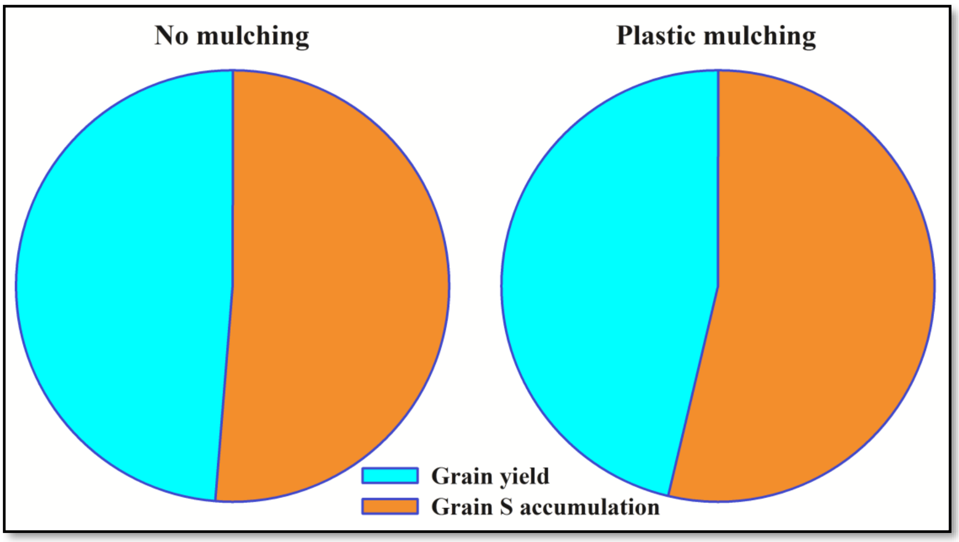

FIGURE S5 Impact of grain yield and grain S accumulation on the contribution rate to grain S concentration of winter wheat in dryland under plastic mulching and no mulching.
